# Supplementary material for: A Computational Model of the Respiratory CPG for the Artificial Control of Breathing
Source: Bioengineering (Basel). 2025 Oct 26;12(11):1163. doi: 10.3390/bioengineering12111163 (PMC12649649; doi:10.3390/bioengineering12111163)
Supplement: Supplementary file 1 [file bioengineering-12-01163-s001.zip › bioengineering-3914855-supplementary.pdf]

SUPPLEMENTARY MATERIALS TO THE MANUSCRIPT:

“A computational model of the respiratory CPG for the artificial control of breathing”

Lorenzo De Toni, Federica Perricone, Lorenzo Tartarini, Giulia M. Boiani, Stefano Cattini, Luigi Rovati, Dimitri Rodarie, Egidio D’Angelo, Jonathan Mapelli and Daniela Gandolfi

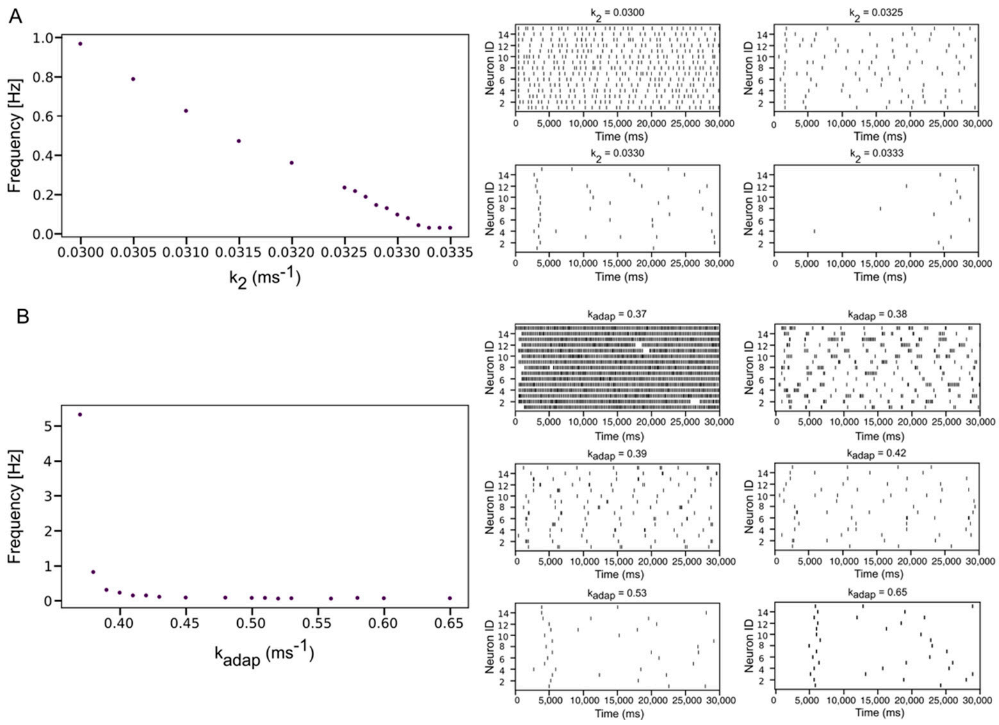

Figure S1. Dynamic regimes as a function of k_2 and k_{adap} parameters. A) Left: Decrease in firing rate with lower k_2 values. Right: raster plots at different k_2 values showing reduced activity as k_2 decreases. B) k_{adap} affects the neuron’s firing regime: low k_{adap} values lead to a bursting regime, while higher values result in a non-bursting regime. Lower k_{adap} values also correspond to a reduced firing rate.

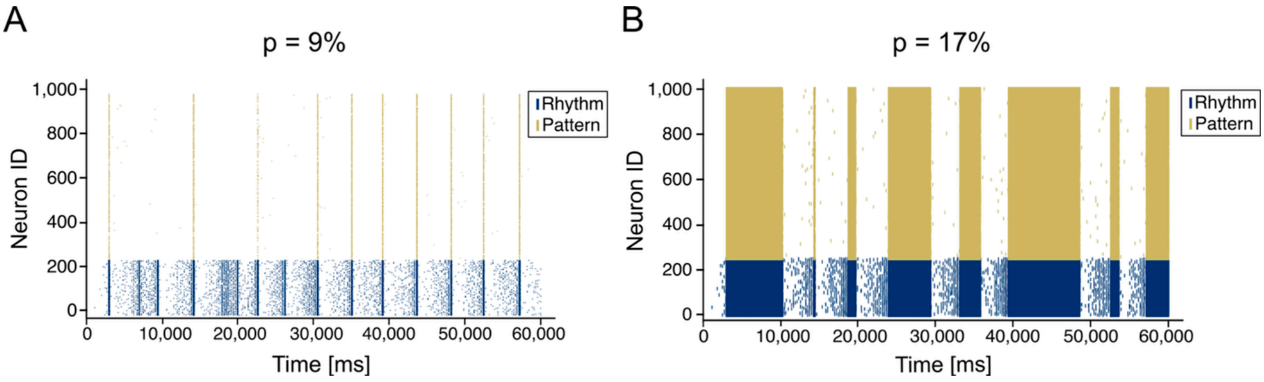

Figure S2. Connection probability. Rhythmic activity is rapidly disrupted in the rhythm population when connection probability is lowered (left 9%) or increased (right 17%).

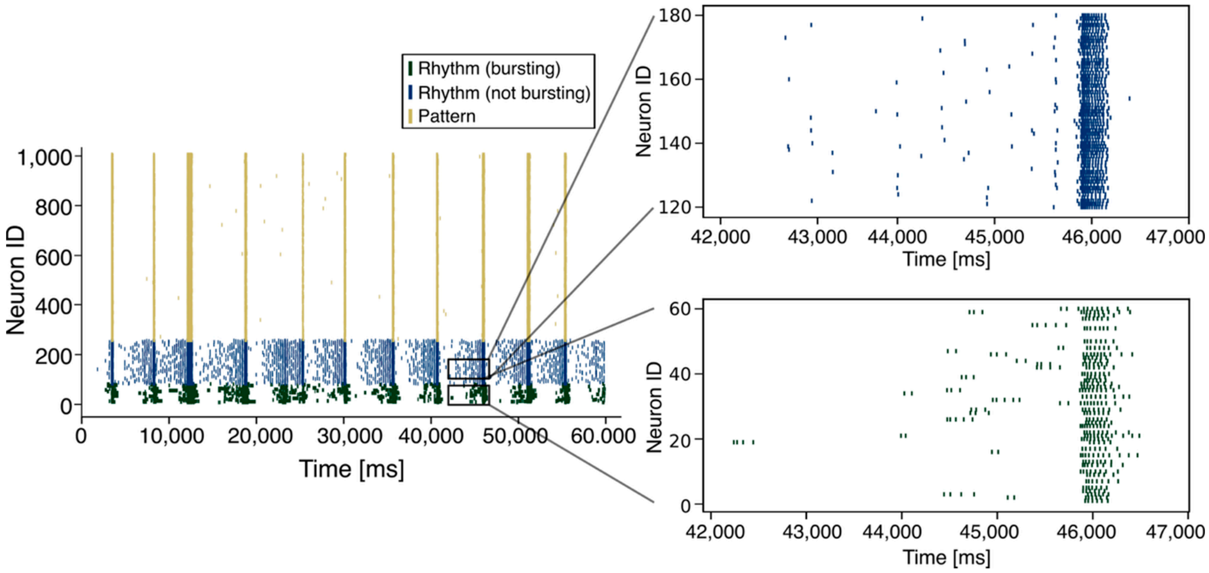

Figure S3. CPG comprising bursting neurons. Rithmic activity is preserved when 30% of the neurons within Rhythm Population are modified to exhibit burst firing. Right insets show the activity of the two neurons subclasses; bursting neurons are shown in the lower panel. The bursting regime was obtained by setting the A_1 parameter to 240 pA, while keeping the other parameters identical to those used for rhythm neurons in the default configuration (table I).

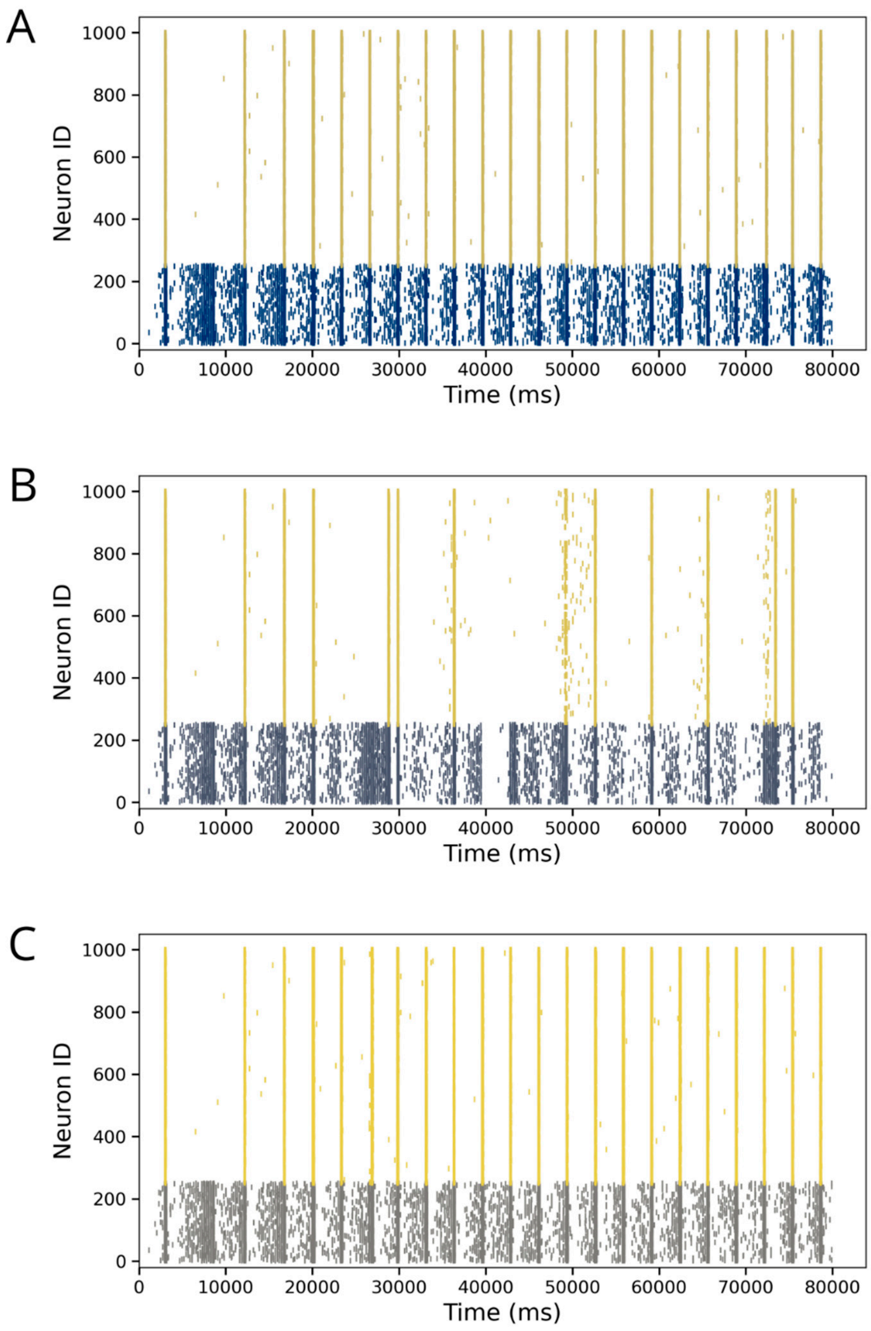

Figure S4. Modulation of preBötC by RTN. A Rhythmic activity of Rhythm and Pattern population when RTN firing is delivered through burst of 3 spikes at 100 Hz. **B** Rhythmic activity of Rhythm and Pattern population is disrupted if RTN when RTN firing is conveyed through single spikes with low synaptic weight. **C** Rhythmic activity pf Rhythm and Pattern population is preserved when RTN firing is conveyed through single spikes and high synaptic weight.
